# Subverting patriarchal narratives: a feminist approach to critical video game design through participatory methods

**DOI:** 10.3389/fsoc.2025.1596630

**Published:** 2025-08-07

**Authors:** Carlos Roberto Torres-Parra, Maria Juliana Florez-Florez, Roberto Cuervo, Omar Fernando Ramírez-Pérez, Oscar Fonseca

**Affiliations:** ^1^Department of Design, Pontificia Universidad Javeriana, Bogotá, Colombia; ^2^Instituto de Estudios Sociales y Culturales Pensar, Pontificia Universidad Javeriana, Bogotá, Colombia; ^3^Department of Communication, Pontificia Universidad Javeriana, Bogotá, Colombia

**Keywords:** critical video games, participatory design, video game design, sexual harassment, feminist pedagogy

## Abstract

This article examines participatory design as a methodology for creating critical video games within the context of feminist pedagogy, focusing on the development of “Poder Violeta 2.” The game aims to challenge sexual harassment experienced by women, feminized bodies, and gender and sexual dissidents in Bogotá’s public transportation system, a context marked by some of the highest rates of insecurity for women. The design process for this new version prioritized the active involvement of women public transport users at all stages and enhanced the use of video game–specific resources, such as multiple playable characters and power-ups, to enrich gameplay and narrative depth. These strategies resulted in greater agency for the game’s protagonists and more accurate representation of historically marginalized groups. Qualitative transformations were observed both in the player experience and within the largely male development team, as the participatory process fostered ongoing learning and co-creation. The outcomes support the value of participatory design in generating inclusive and representative video game content, highlighting the narrative and pedagogical potential of medium-specific elements while emphasizing the empowerment and participation of historically excluded communities as a reference for future projects.

## Introduction

**Figure 1:**
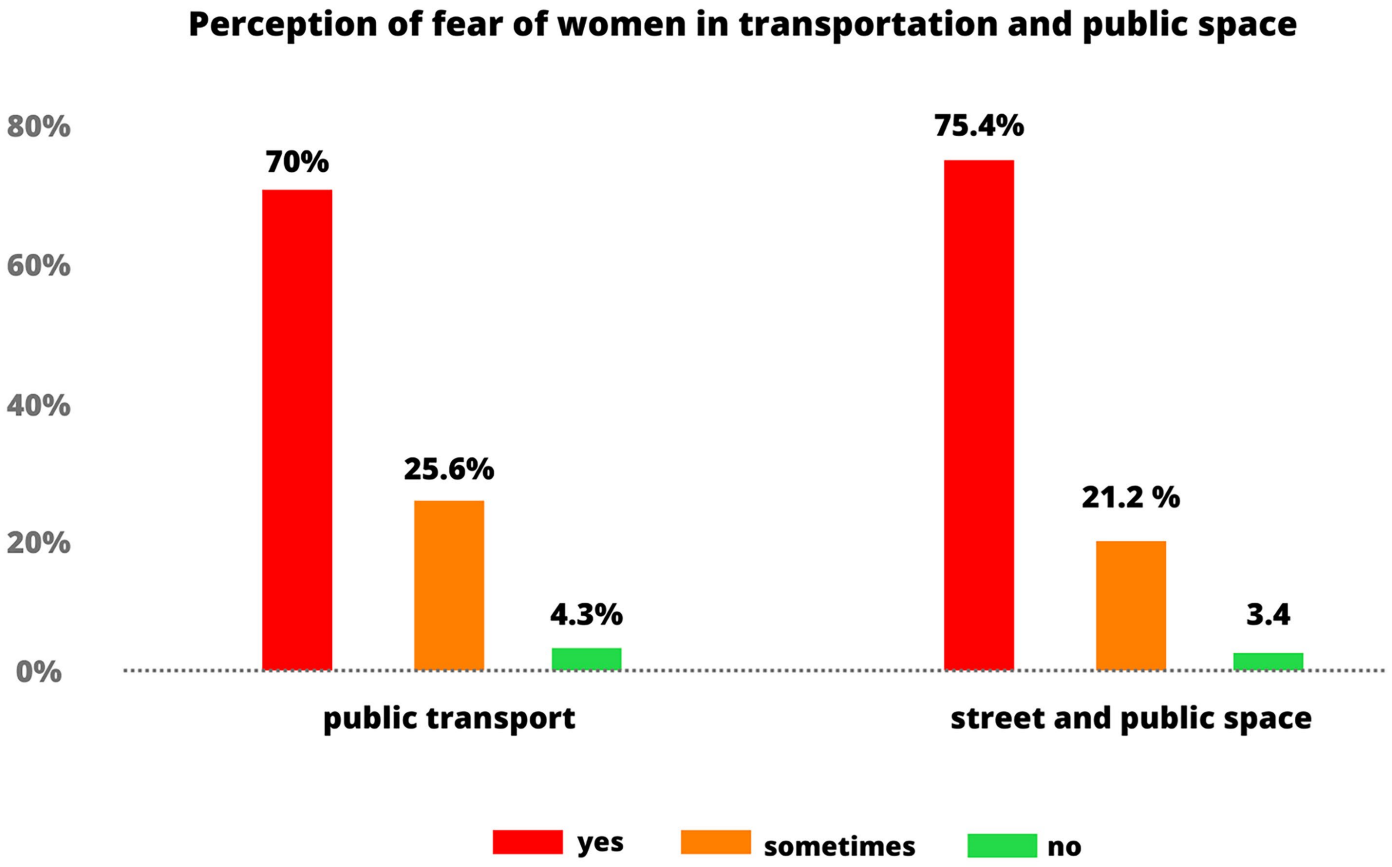
Perception of fear among women in transportation and public space. Based on graphs from Veeduría Distrital de Bogotá (2022).

Sexual harassment poses a serious contemporary social problem within public transportation, particularly in low- and middle-income countries where most women have encountered some form of sexual violence (Irvin-Erickson and Gurvis, 2017). For example, in Bogota, a survey conducted by the Veeduría Distrital de Bogotá (2022) revealed that seven out of 10 women experience fear of sexual assault in public transportation due to inadequate security and a culture of impunity (Figure 1). The problem worsens as victims frequently choose not to report such incidents due to feelings of indifference, neglect, fear of judgment, or guilt. One factor in the problem is the lack of effective policies to prevent harassment and protect victims, but this and other previous research identify socially ingrained harmful ideas about the nature of men and women’s behavior as a frequent cause (Rahman et al., 2022).

The lack of education and awareness on the subject is also concerning, so this project focuses on the cultural dimension of this phenomenon.

If we understand culture as a set of values, norms, beliefs, practices, and symbols that shape social behavior (Geertz, 1973; Bourdieu, 1977), it is pertinent to consider the role that widely consumed products like video games can play in this process. As a dynamic and evolving entity, culture offers a space to reflect on how video games—viewed as cultural artifacts—can become powerful tools for conveying alternative narratives to those typically found in mainstream media, thereby contributing to social change (Torres, 2013; Denisova and Cook, 2019; Shliakhovchuk, 2024). From this perspective, critical video games form a genre intentionally designed to question dominant narratives and power structures by engaging players with issues such as gender-based violence, racial justice, and discrimination (Frasca, 2007; Flanagan, 2009). Unlike games focused solely on entertainment, critical games leverage gameplay and narrative as means of social critique and transformation. A key theoretical foundation for this work is procedural rhetoric (Bogost, 2007), which refers to the way video games persuade or communicate ideas through their rules and mechanics rather than just through text or visuals. This means that the game’s design itself embodies arguments or perspectives that players experience interactively.

The video game “Poder Violeta 2” is understood as a cultural artifact, similar to expressions such as literature, painting or cinema, which has the potential to mobilize discussions and reflections around complex social issues, in this case, sexual harassment in public transportation. The intention is not that the video game alone will cause a direct and measurable change in men’s behavior or an immediate reduction in incidences of harassment, but that it will generate a space for critical reflection and dialogue among its players, thus contributing to influence culture and, eventually, social behaviors. This research adopts a critical design trend that designs video games as spaces of intense socialization that can either recreate power relations or question them. These games often focus on themes such as racial justice, gender violence, online harassment, and the fight against discrimination. Additionally, initiatives have employed certain games to raise awareness about environmental issues and foster a sense of solidarity and mutual aid. These so-called “critical” video games demonstrate how this medium can be a powerful tool to promote social change and foster a more just and equitable culture (Frasca, 2007; Flanagan, 2009, 2023; Flanagan and Nissenbaum, 2014; Torres, 2015).

As in the first version of Poder Violeta (Purple Power), Participatory Design was used to engage those directly affected by the issue, gathering insights on both the problem and possible solutions while fostering collective agreements to validate the proposed designs. This methodology involves users and stakeholders directly in the design process. By integrating those affected by the issue-here, women and gender-diverse public transportation users-participatory design aims to create more relevant, inclusive, and empowering games while fostering collective ownership of the final product (Kyng, 1991; Torres et al., 2022).

In the context of video game design, involving stakeholders in the process helps strike a balance between content and gameplay, reducing biases and encouraging creative innovation (Torres et al., 2022). However, as will be demonstrated in this iteration, this approach has been further deepened to enhance its effectiveness.

Poder Violeta 2 was the response to evaluations, questions and discussions carried out by feminist university collectives in Bogotá on the first version of Poder Violeta, which was a game for mobile devices on Android and iOS platforms. The plot follows Violeta, the protagonist, who needs to arrive punctually for a job interview, having to evade aggressive harassers, typical in public transportation during her journey. After evaluation, the second version of the game prioritized actively involving its protagonists in the design stage, addressing the shortcomings identified in the previous iteration. To achieve this, the team delved deeper into the participatory design method, encompassing all stages of video game creation, with a particular emphasis on the “translation” stage, that involves transforming conceptual ideas and social theories into concrete game mechanics and narratives. This paper aims to present the development process of Poder Violeta 2, highlighting formal and mechanical aspects that contribute to what Ian Bogost (2007) refers to as the “rhetoric of video games,” which can effectively convey a message and represent phenomena like those addressed in this game. Lastly, we reflect on the potential of critical video games to raise social awareness and foster social change.

## Materials and methods

### Critical video games

This approach to video games design emerged in the early 21st century, tied to activism, social transformation, and the denunciation of injustices (Dyer-Witheford and De Peuter, 2009; Flanagan, 2009; Flanagan and Nissenbaum, 2014). Recent initiatives highlight video games’ potential to drive environmental and social change (Stanitsas et al., 2018; Patterson and Barratt, 2019). Flanagan and Nissenbaum (2014) highlight that video games are not just entertainment but powerful tools for social reflection, embedding values into their design. This connects to how cultural products shape norms and behaviors. Bogost (2007) reinforces this through procedural rhetoric, which persuades players via rules and mechanics. The Playworld matrix (Frasca, 2007) expands on this by incorporating symbolic elements alongside rules. Building on these ideas, Torres et al. (2015) introduces the CTV cyclical design scheme: Conceptualization, Translation, and Verification, which will be explored further.

The video game industry remains concentrated in a small number of countries. As a result, and despite its potential, the production of critical, not-for-profit games is concentrated in the wealthier nations, posing significant challenges for the sector in the Global South.

In addition to foundational theorists such as Bogost and Flanagan, this project draws on key contributions from feminist game studies scholars Shaw (2014), Gray (2014), and Chess (2017). Shaw’s work on gender representation challenges normative portrayals in gaming culture, while Gray’s research on race and intersectionality informs a nuanced understanding of marginalized identities within digital spaces. Chess’s feminist critiques emphasize the political stakes of representation and support participatory, inclusive design approaches. Engaging with their scholarship strengthens the project’s theoretical grounding and aligns it with current debates on diversity, equity, and inclusion in video game design.

### Sexual harassment in public transport: a contemporary and serious problem

Feminist critical theory has conceptualized sexual harassment as a specific form of gender-based sexual violence that is commonly experienced by women, as well as feminized and gender non-conforming bodies within the system of sexual heteronormativity. According to Quiñones (2020), this experience involves a set of violent practices, typically perpetrated by men that elicit sexual activity against the will of those who suffer them. Additionally, the author argues that sexual harassment can take on several forms, such as verbal (catcalling, lewd expressions), physical (rubbing, cornering, and other forms of physical contact), or visual (lewd glances).

This is a serious contemporary social issue affecting both wealthy and poor countries. However, the fear of experiencing sexual assault in public transportation or public spaces disproportionately impacts women in middle- and low-income countries. Studies focusing on these regions have shown that nearly 90% of women have encountered some form of sexual violence, particularly within public transport systems (Irvin-Erickson and Gurvis, 2017). It is important to clarify that this statistic is not global but reflects data primarily from middle- and low-income countries. Despite its prevalence, the true scale of this phenomenon remains unclear, especially in Latin American cities.

In Buenos Aires, a report by the Marea group in collaboration with the Popular University Pie (2023) revealed that six out of 10 women surveyed reported instances of sexual harassment while using public transportation (Buenos Aires Times, 2023). The situation in Bogotá is even more concerning. A 2022 government survey found that 7 out of 10 women fear sexual assault in public spaces, and 8 out of 10 have experienced sexual harassment—an increase compared to 2018 (Despacio, 2018). Furthermore, in 2014, the Thomson Reuters Foundation ranked Bogotá as having the world’s most dangerous public transportation system for women (Boros, 2014).

Victims often avoid reporting violent incidents due to indifference, negligence, or fear of judgment. They may also feel guilty, as society often blames them for provoking harassment, particularly based on their clothing (Quiñones, 2020). Another issue is the tendency to design public policies, including urban and transportation development, around men’s experiences of the city (Fenster, 2005).

Sexual harassment is rooted in the patriarchal system and intersects with other forms of oppression, including racism, classism, and sexual heteronormativity. From an intersectional perspective, these overlapping systems of oppression intensify the violence experienced by feminized bodies, particularly affecting Black, poorer, and trans-feminized individuals who face heightened vulnerability in public transportation spaces (The Combahee River Collective, 1977; Crenshaw, 1991; Hill Collins, 2000). Recognizing these intersecting identities is essential to understanding the varied and compounded experiences of harassment and to designing more inclusive and effective interventions.

The project highlights sexual harassment in Bogotá’s public transportation as a key theme for the video game developed in this research, due to its high frequency, varied forms, and complex nature. The game aims to raise awareness about the issue and create spaces for critical reflection on systemic violence, encouraging players to view sexual harassment in public spaces as unacceptable.

### Target population

The game is targeted primarily at young males, especially within the 15–29 age range, which corresponds to the demographic group most active in the consumption of casual video games on mobile devices. This decision responds to the evidence that most perpetrators of sexual harassment on public transport are young men, according to research cited in the article itself and in the literature on gender-based violence 1 (Boros, 2014). Therefore, the main objective of the game is to intervene preventively in male socialization, promoting critical reflection on harassing behaviors and fostering empathy toward the victims.

By focusing the game on young men, we seek to influence a key stage in the formation of values and behaviors, using a medium (casual video games for cell phones) that is familiar, accessible and attractive to this population segment. This orientation does not exclude the participation of other audiences but prioritizes young men because they are the ones who, according to statistics and studies cited in the paper, most frequently reproduce harassment behaviors in public spaces. Thus, the game becomes a pedagogical tool aimed at transforming imaginaries and practices from the root of the problem.

### About the first version of Poder Violeta and its limits of representation

**Figure 2:**
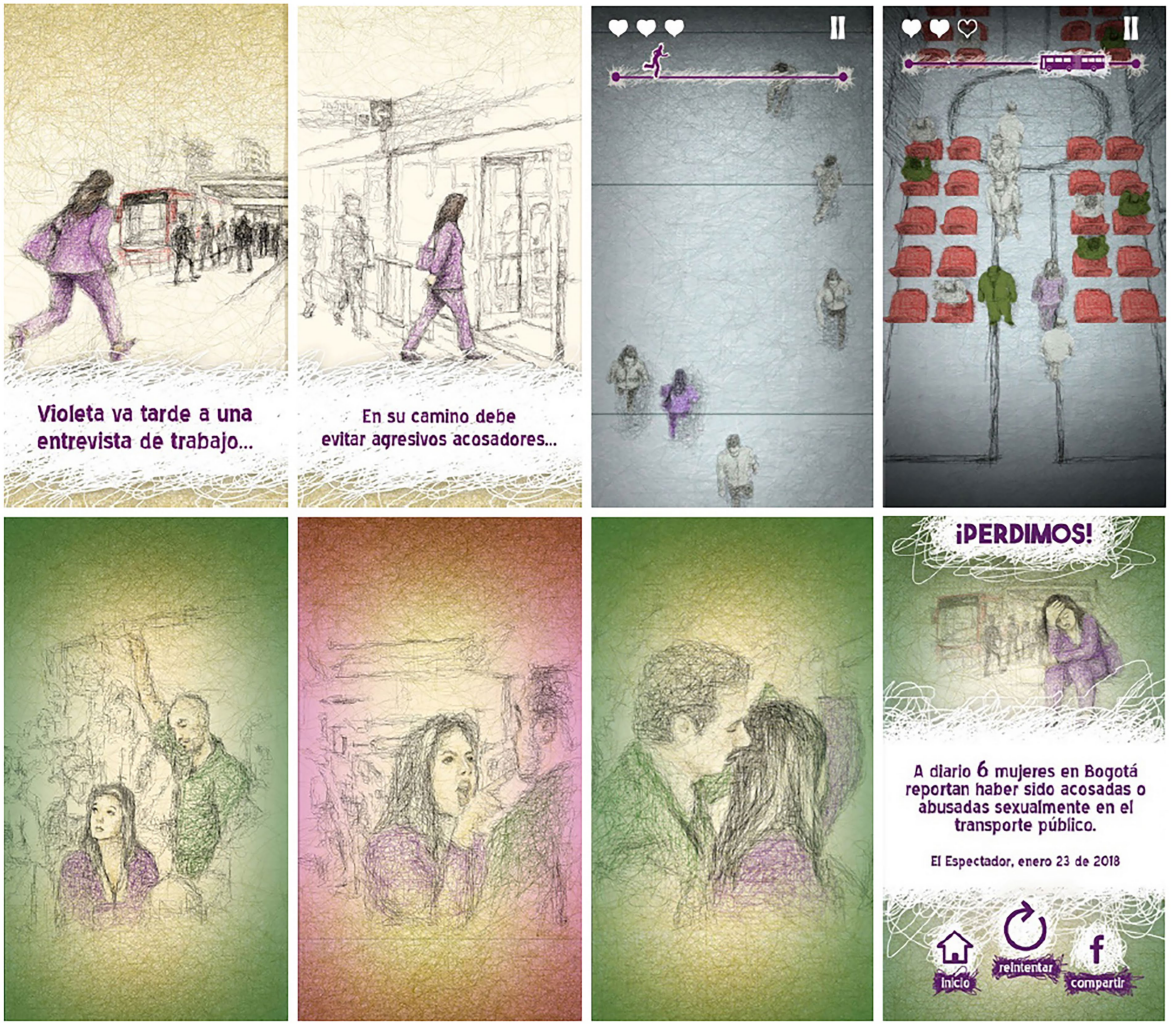
Images from the first version of the video game “Poder Violeta” [Reproduced from Torres et al. (2022), licensed under CC BY-NC].

In 2018, the researchers designed the mobile game “Violet Power” (Figure 2) using the CTV creative scheme in a participatory design approach (Torres et al., 2022). Like many video games, its creation required the convergence of multiple disciplines, including visual and sound arts, design, and programming (with a focus on critical game studies, feminist critical theory, and participatory methods), through the implementation of various audiovisual resources into an interactive artifact. The main objective of the game was to represent a simple dynamic in which Violeta, a young woman living in Bogotá, must arrive on time for a job interview while avoiding sexual harassment in different stations and vehicles of public transportation.

The project garnered a positive reception in various Latin-American countries (Ciriaco, 2018; Canal Trece, 2018; Del Real, 2018; Tupper, 2018; Wong, 2018). However, during the verification phase, some feminist university collectives in Bogotá argued that while the video game succeeded in making sexual harassment in public transportation visible, its design had two serious problems: first, representing those who suffer violence in a passive manner and second, recreating a game mechanic that always leaves the protagonist at a disadvantage. Both limitations diminish the critical potential of the video game and, in part, result from not having deepened the participation of the protagonists of the problem stated in the verification stage. As a result, their observations were unable to inform adjustments and modifications in a second design phase. Hence, there is the need for a new version that would deepen their participation in all stages of the design.

On the other hand, it was observed that the game primarily focused on the experiences of women who conform to conventional beauty standards and dress codes, overlooking the reality that women of all appearances and clothing styles can be victims of sexual harassment. Additionally, it exclusively depicted male harassers and female victims, disregarding the possibility of same-sex harassment and the victimization of non-binary individuals. The game also failed to address the intersectionality of oppression, neglecting how factors such as race and socioeconomic class influence experiences of sexual harassment in public transportation.

Furthermore, during the testing phase, several players reported that the game’s pacing was too slow and its complexity too low, which made the experience less challenging and at times not engaging enough as a gameplay obstacle (Torres et al., 2022). This slow rhythm reduced the sense of urgency and tension that could better represent the protagonist’s struggle to evade harassment, thereby limiting the critical impact of the game’s mechanics. We revisit this issue later when discussing gameplay design improvements.

Despite these limitations, “Poder Violeta” still represented a significant step forward in using video games as a means of addressing social issues such as sexual harassment. Its use of interactive media allowed players to experience and understand the impact of sexual harassment in a unique and immersive way. Nevertheless, it is important to recognize the limitations of this representation (Figure 2) and strive for greater inclusivity and intersectionality in future versions of the game.

### Participatory design methodology

While participatory design is often associated with the politicization of design championed by the Scandinavian labor movement in the 1970s (Bjerknes and Bratteteig, 1995), it is important to acknowledge, as Udoewa (2022) points out, that participatory approaches have existed in communities for millennia and are sometimes considered “ancient.” As can be intuited, this way of designing consists of developing products or services integrating those who will be their users (Kyng, 1991). The pragmatic intention of involving people without design training in the creative process is precisely to obtain better designs.

Recently, participatory design has been recognized as particularly valuable for critical perspectives aimed at fostering social transformation (Lee, 2008), promoting democratic experiences and social progress (Manzini, 2015), and challenging contemporary power relations (Kraff, 2019). Despite the growing presence of critical approaches in video game development, the adoption of participatory design methods in this field remains limited (Isbister et al., 2010; Khaled and Vasalou, 2014; Gerling et al., 2016; Ampatzidou and Gugerell, 2018). Participatory design can enhance communication between individuals with complex needs and those seeking to represent them through designed artifacts, as exemplified by video games developed by Hart et al. (2020).

Moreover, Malazita and O’Donnell (2023) argue that incorporating participatory design in video game development can amplify marginalized voices. In particular, they highlight early examples of how feminist, Indigenous, and queer approaches have demonstrated their potential to challenge and transform the discipline from within.

Following this emerging trend, both versions of Poder Violeta used participatory design as a powerful tool to develop video games that stimulate conversations about various social problems. Through this form of design, people affected by injustices can be an active part of the design process, ensuring that their perspectives and needs are at the center of the project.

For this project, the participatory design methodology was deepened and applied across all stages of video game development to ensure that the creative process includes the perspectives of those directly impacted by the issue at hand. Drawing on Flanagan (2009) and Flanagan and Nissenbaum (2014) work on games as tools for social reflection and transformation, participatory methods actively involved women at every stage. This collaboration ensured that characters and narratives reflected authentic experiences rather than passive or stereotypical portrayals. For instance, protagonists were given greater agency through character development and power-ups symbolizing empowerment, enriching both gameplay and its potential for social impact. For this purpose, the project called for university students from Bogotá who used public transportation daily and belonged to a university feminist organization whose agenda included the problem of sexual harassment. Thirty participants, mostly belonging to a university feminist organization, were selected through non-probabilistic sampling of typical cases. It was non-probabilistic in that the selection was guided by the characteristics of the research (belonging to a feminist organization), rather than a statistical criterion of generalization. In addition, there were representative cases because it focused on their experiences with sexual harassment and as activists of their organizations, both criteria provided greater richness and depth to the research. Participants were not paid for their involvement in the development of the game. The project followed ethical guidelines grounded in participatory design philosophy, emphasizing voluntary participation and respect for the contributors’ experiences and voices. This approach sought to create a collaborative and safe environment where participants could freely share insights and co-create the game without coercion or financial incentive.

## Results

Poder Violeta 2 was launched with the same objective as the first version: to generate questions in young people about the sexual harassment that women, feminized bodies and sexual and gender dissidence commonly face in public transportation. It also followed similar creative parameters as the first version, in terms of the immersive soundscape, visual style and development software. However, unlike the previous version, the new version applied participatory design in greater depth in all creative phases and applied concepts about the variety of victims who suffer harassment and dynamics to confront it in some formal elements of the game, such as the variety of characters and their powers, incorporating them into the game mechanics. The following section presents the analysis of the results, following the three-phase CTV cyclical scheme.

### Conceptualization

In this phase, the design team identified the problem elements to be addressed by the video game, as well as the relevant agents and dynamics. This identification process involved a guided session using the design concertation technique, which facilitates collaborative dialogue and consensus-building among stakeholders during the design process. In this case, participants were guided through sessions in which they actively engaged in discussions, negotiations and alignment of their perspectives to define the problem. Dialogue with feminist critical theory was also crucial in this process. As a result, the following was defined:

- Agents: identified as harassers, victims, and bystanders, the process exposed the representation limits of the first version of the video game, such as the representation of the character of Violeta under parameters of sexual heteronormativity and the passivity of the protagonist’s response that leaves her at a disadvantage in the situation of harassment.
- The dynamics: the team agreed on specific design solutions to remedy these constraints (to achieve fewer passive expressions of the character, diversify her representation, design powers that do not leave her at a disadvantage, and enrich the game’s dynamics).
- The elements of the problem: the types of sexual harassment that the video game should express were refined. In addition to those contemplated in the first edition (physical, verbal, visual), the sound type was included.

### Translation

In this second creative phase, the aim is to employ video games as a means of communication, involving the portrayal of the problem using the language specific to video games. To achieve this, it is essential to identify and utilize the attributes that enable this medium to convey a message, such as symbolic elements like characters, settings, texts, and sounds, which are common in other forms of audiovisual expression. Additionally, it is necessary to consider the rules that govern the operation of these elements, a crucial aspect of games that is rooted in their interactive nature (Flanagan, 2009; Bogost, 2007). In Poder Violeta 2, the gameplay requires players to evade harassers, simulating the real experiences of victims and enabling players to feel vulnerability and resistance interactively. This approach ensures the game’s design embodies its critical message through gameplay rather than relying solely on narrative or visuals.

**Figure 3:**
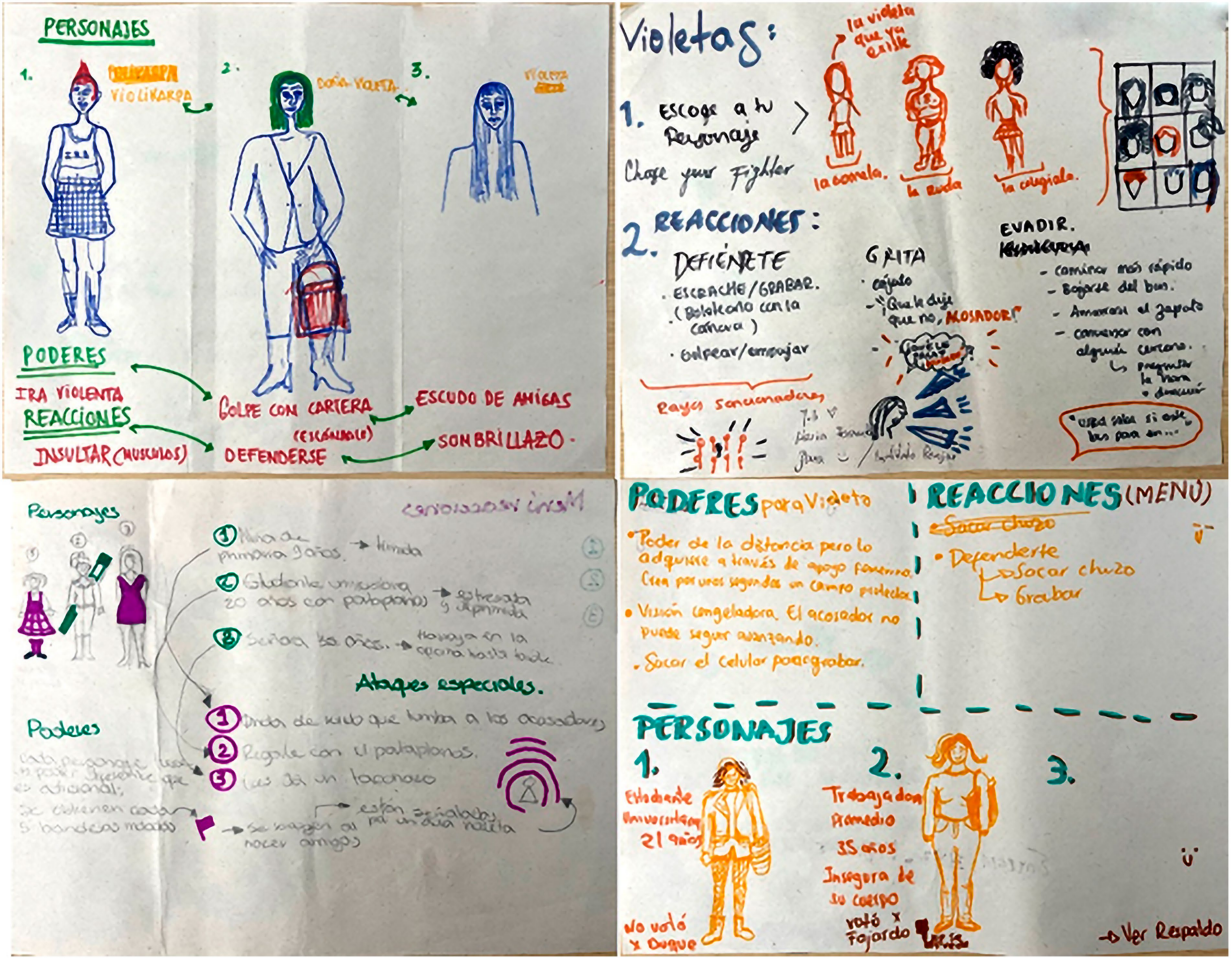
Some character design proposals from the workshops.

Unlike the first version of the game, this stage required a deeper creative process, which led to the development of six workshops. Using a collective drawing technique, participants created a range of characters representing different types of harassment victims. Through group discussion and consensus, these were refined into five versions of the protagonist. The visual proposals were then discussed, refined collectively. This process fostered active participation, creativity, and consensus-building, ensuring that the game’s characters and mechanics reflected the participants’ perspectives and lived experiences (Figure 3).

**Figure 4:**
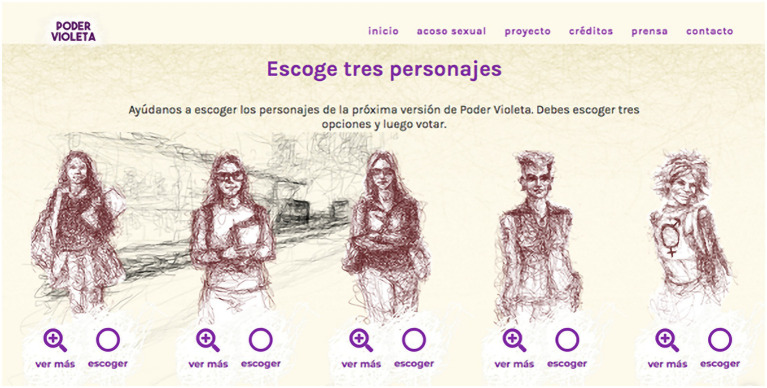
Five portrayals of the character of Violeta.

In this project, a professional artist interpreted these drawings in a technique that displays the strokes of the drawing, contributing a unique aesthetic that contrasts with the typical imagery of video game graphics. The illustrations incorporated a diverse range of appearances, ages, occupations, aesthetics, etc., seeking to provide a more heterogeneous representation of women, feminized bodies, and sexual and gender dissidents who are victims of sexual harassment in public transportation (Figure 4).

From left to right:

Violeta 1. She is a 13-year-old high school student in seventh grade and spends 2 h a day on public transportation - Transmilenio - to go from her home to school and another 2 h to return. She can activate her paralyzing wave when bullies are nearby to defend herself.

Violeta 2. Is a college student and part of the Polygender collective. To get to her 7:00 am class, she must take two transfers on *Transmilenio*. Her power is the luminous call-out that quickly allows her to point out to the other passengers the harasser who is intimidating her.

Violeta 3. A precarious worker in a “call center,” with an 11-h workday. She must get home early to take care of her cat, Pancho. When she activates her power, nearby harassers feel the strength of her circular blows delivered with her handbag (carterazos).

Violeta 4. Plays bass in a punk band, loves music, and every afternoon she must hurry to pick up her daughter from school. She has a purple ray that dissipates harassers.

Violeta 5. Is a transexual activist in organizations supporting sexual diversity. Her power is a scream that generates a supersonic wave capable of pulverizing harassers.

As can be seen, Crenshaw (1991) theory of intersectionality is translated into the game design through the inclusion of characters and interactions that reflect overlapping identities and oppressions. In Violet Power 2, not only women appear, but also, trans, and lower-class people, illustrating how intersecting identities increase vulnerability to harassment on public transportation.

In addition to each character’s own powers, the game includes other “powerups” into the game’s dynamics, available to any of them: the game provides characters with a shield that grants them temporary protection and the ability to turn invisible for a limited time, allowing them to move forward undetected by harassers.

Following the same logic as the first version of the game, the player must reach their objective (the bus entrance when it is at the station or the exit door when it is on the bus), while avoiding various types of harassment. Violeta must arrive on time and without the harassers depleting her energy. To enhance the game experience, the development team created both general and specific power-ups in addition to the characters. Power-ups are integral elements in video games that enhance player experience by providing temporary advantages or altering gameplay mechanics. They serve as rewards that can significantly influence player engagement, immersion, and overall satisfaction. These elements can include a variety of forms such as extra lives, improved weapons, protective shields, increased speed, among others. To obtain power-ups, players must interact with specific objects or achieve certain objectives within the game. They often appear in the form of icons or specific elements in the game, can be temporary, which means they disappear after a certain time, or permanent (Gee, 2003). Research shows that power-ups enhance player experiences, boosting immersion and autonomy. Notably, even “placebo” power-ups, with no real gameplay impact, evoke similar feelings of engagement (Denisova and Cook, 2019). The team used this resource aimed to overcome the passive representation of Violeta in the initial version of the video game, providing the character with more action capabilities within the situations represented in the video game.

As previously noted, Flanagan’s (2009) theory of *critical play* guided the project by framing it as a form of subversive design, in which video game mechanics can serve activist and pedagogical purposes. Through the lens of *procedural rhetoric*, as defined by Bogost (2007), which translates rules and behaviors into meaningful systems, *Poder Violeta 2* invites players to experience and critically reflect on gender-based violence and public harassment, while also encouraging strategies of resistance.

Furthermore, the incorporation of several concepts exposed in feminist game studies into the game’s dynamics enriched the overall experience. The mechanics, enhanced by the implementation of special powers and power-ups, along with character diversity and carefully modeled interactions, highlight forms of resistance by individuals subjected to violence, interpreted through the lens of intersectional theory. As previously discussed, Crenshaw’s (1991) concept of intersectionality is directly reflected in the game’s diverse cast of playable characters, each of whom embodies overlapping identities and systemic oppressions such as gender, race, and social class.

The dynamics of sexual harassment translated into the game mechanics and design elements of the game include various forms of aggression experienced by women in public transportation, such as verbal catcalling, physical rubbing, cornering, and intrusive visual contact. These behaviors, drawn directly from participants lived experiences and narratives, are embodied in gameplay through obstacles and antagonistic characters that the player must evade or confront. The game’s mechanics simulate the vulnerability and constant negotiation of space that victims endure, while power-ups and multiple playable characters provide agency and resilience, reflecting diverse identities and intersectional vulnerabilities.

Shaw (2014) argues that video game content is never neutral but always embedded within specific social and cultural contexts. Her critique of the dominant representation of white, heterosexual, male characters in games highlights the importance of featuring protagonists in Poder Violeta 2 who reflect the real-world diversity of both players and victims. This aligns with the game’s goal of subverting hegemonic tropes and, as detailed further in the manuscript, advocating for more inclusive representations within the video game industry.

Similarly, Gray (2014) shows how whiteness and masculinity operate as normative forces within gaming environments, labeling non-conforming bodies as deviant. Her reflections resonate with various design decisions aimed at making marginalized identities visible not only visually, but also procedurally, through the powers, roles, and interactions assigned to each character. A sound landscape represents the auditory environment of an action or event, including both natural and artificial sounds. For Poder Violeta, a team member recorded the public transport soundscape using a high-quality ambisonic recorder to create an immersive experience. The sound was then edited in a studio to produce a surround sound environment. Additionally, the game included intrusive, irritating sound effects for the harassers’ voices, enhancing the feeling of harassment and increasing player immersion.

Following the design of the five characters, the team launched an online voting campaign on the project’s website, enabling potential players to engage in the decision-making process for a month. Thus, the public was invited to choose three out of the five-character options, so that the three most voted versions of Violeta could be incorporated into the final version of the game. With around 1,800 votes, the highest vote was for the school student (25%), followed by the worker (20%) and finally the musician (19%).

### Verification

**Figure 5:**
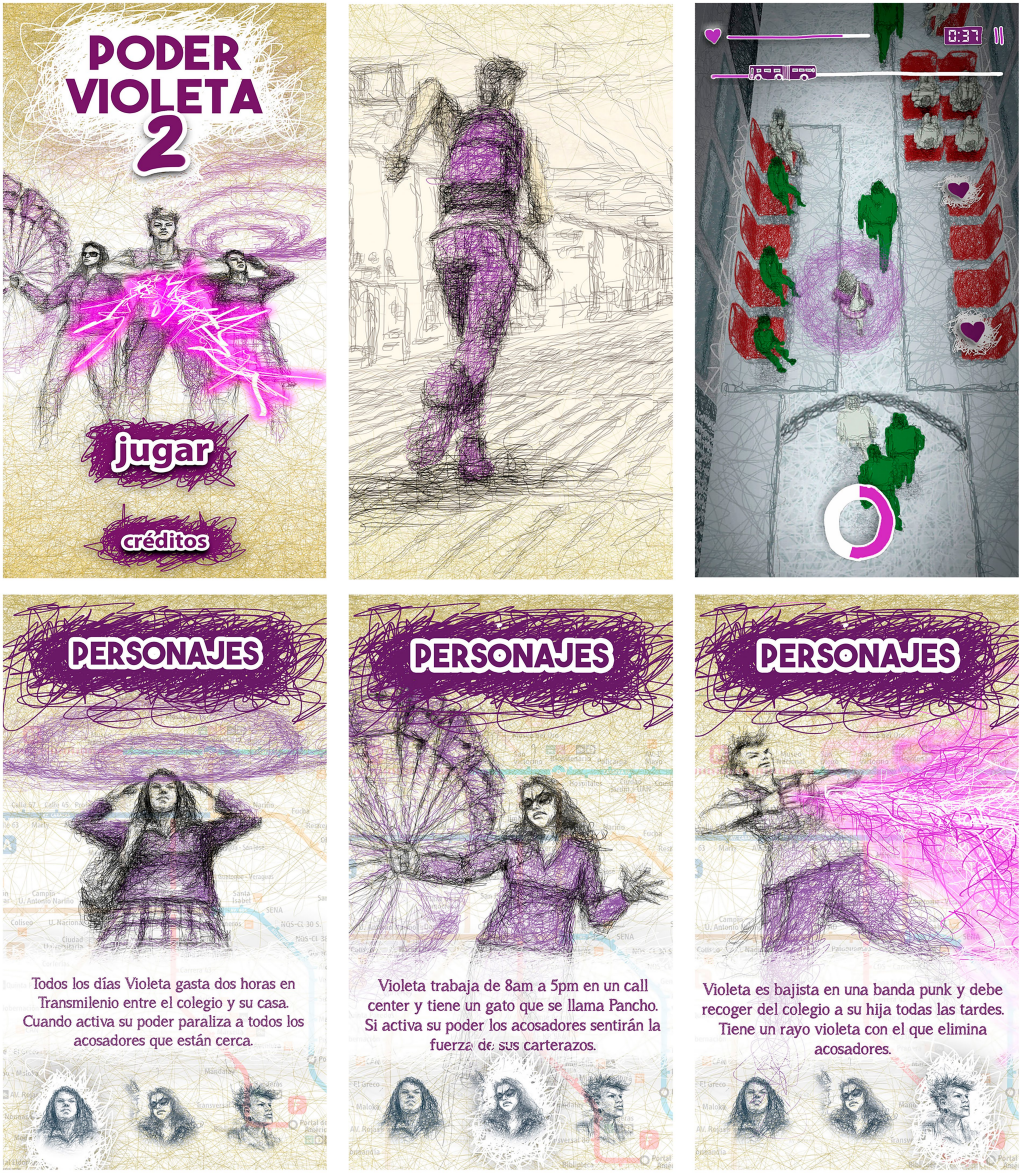
Final images of the video game.

During this final stage, the evaluation and refinement of the proposed idea involves assessing not only functional aspects but also the interpretation of the concept (Figure 5). To do this, three activities were developed:

Open game session: to identify potential flaws in the game’s operation, the project team organized and recorded a virtual session. Seven people from the project’s target population participated in the session. Based on the recordings, adjustments were made to balance the game, calibrating the difficulty of the first levels to be less challenging and facilitate player progress. Additionally, we adjusted playability, which is related to the characteristics that allow players to interact with and control the game. Good playability considers control mechanisms and game dynamics. Playability is essential for a satisfactory gaming experience because it gives players effective control over the game and allows them to experience a sense of achievement. After testing, the developers adjusted the game’s mechanics, rhythm, and difficulty to create smoother gameplay in the two scenarios where the action takes place (stations and buses).

Questionnaire: To assess the design process, the outcome, and its capacity to initiate discussions on sexual harassment, the project team distributed a questionnaire to 25% of the participants in the creative workshops.

The responses to the question “Compared to other video games you know, do you think Poder Violeta can open discussions about sexual harassment, and if so, why?” coincide in highlighting the game’s potential to generate reflection and dialogue about sexual harassment. Several participants pointed out that Poder Violeta clearly and directly represents everyday situations faced by women, which facilitates recognition and identification with these experiences. As participant A stated: “Yes, because it shows graphically how, where and why situations of sexual harassment of women occur.”

In addition, it is valued that the playful format allows attracting diverse audiences, including people who are not familiar with feminism or with the issue of harassment. This broadens the scope of the discussion beyond the usual circles. It also points to a “double track” of reflection: not only on harassment in the public space, but also on misogyny in digital environments themselves. In the words of participant B, “Both about street harassment, which is their target, and about harassment in digital spaces because the world of video games is very misogynistic.”

Finally, some responses highlight the empathic value of the game, which allows to experience indirectly the impact of bullying from an emotional perspective. Overall, the opinions show that Poder Violeta is perceived as an expressive medium with critical and transformative capacity, capable of generating reflection and conversation at different levels.

- Open virtual forum. The project reached out to different collectives and feminists, actively involving them in showcasing the video game, fostering an inclusive event that allowed for a range of viewpoints to be expressed. While the initiative received favorable reception, some participants questioned the predominance of a male team in the video game’s design, as will be explored in the conclusions.

### Reception

Poder Violeta 2, like its first version, was well received, with its creative process (Figure 5) even being featured in local press (Cortés, 2020). The video game has several positive reviews on the platforms where it is published and has been highlighted twice by Apple as one of the best applications in the App Store in Colombia.

## Discussion

Video games are a cultural product that can reflect the values, tastes, and aspirations of the societies in which they are created, including their injustices. However, it is important to note that these cultural elements are dynamic and subject to change over time. An innovative approach in video games could challenge these unfair trends, drawing young players’ attention to the more complex aspects of our society and questioning gender and sexuality representations that are often present in this popular entertainment medium.

If we consider that digital media influences how our society behaves, video games can be a powerful tool to resist different forms of domination, including those related to gender and sexuality. In fact, it could become a transformative device for these relationships. To achieve this, it is necessary to articulate new action frameworks in which young people, who are regular consumers of these contents, relate to fairer narrative discourses and discover different stories that question, above all, patriarchy. This project offers an alternative to quantitative research methods that measure behavioral change in video game players. While such methods are understandable, they are not typically used for other cultural expressions like cinema, literature, or music. The project focuses on the cultural aspect of video games, encouraging players to reflect on a specific issue.

On the other hand, incorporating participatory design methodologies in all stages of the development of a critical video game strengthens the enunciation of the message that the game wants to convey. Participating in these types of processes not only involves intervening in the design of a product that will later be used but also promotes more reflective communication practices about hegemonic representations of gender and sexuality.

In the case of the development of the video game “Poder Violeta 2,” three notable advances stand out when comparing them to the first version. At a strictly playful level, the game became richer by increasing its complexity by incorporating more characters, powers, and “power-ups.” Based on the findings of the verification phase, it becomes evident that the game is more dynamic. As participant C stated: “More levels, more powers, more interaction and paths within the video game. A more realistic appearance of women, various situations in which harassment takes place.”

Second, in the realm of organizing frameworks of action, aesthetics, and ethics, with the new version of the videogame, the character gained more diversity in the representation of those who suffer sexual harassment in public transportation, as well as greater room for maneuver in their ability to respond to the reported problem. As explained by participant D: “[.] adding powers and characters added complexity to the moment of showing harassment. On the one hand, it indicated that several types of women have to deal with this, and on the other hand, it made Violeta have a more direct way of facing situations, revealing her agency.”

Third, the design process ensured a more active role for the protagonists of the video game. As participant E indicated: “We helped think it from the beginning. My classmates and I tried to make the video game as intersectional as possible, to qualify the scope and limits. Talking about our own experience allowed us to broaden the perspective.”

Despite the advances achieved in the development of the video game “Poder Violeta 2,” the project generated new issues regarding the legitimacy of a critical video game considering the identity of those who developed it. Participants expressed concern about the predominant male composition of the team responsible for the video game development, highlighting its problematic implications for representation and diversity. This was discussed throughout the design process and arose in the open virtual forum, where the team’s ability to express the issues sought to be addressed from a more inclusive and diverse perspective was questioned.

This imbalance required the participants, in addition to contributing design ideas, to engage in feminist pedagogy with the development team. As participant F indicated: “There were many discussions and there was always surprise among them around the anecdotes of harassed and assaulted women. Surely it was also a shock for them to encounter our positions.” Unfortunately, from a technical development perspective, this project mirrors some aspects of the video game industry’s dynamics. The field remains male-dominated, with women making up only 23% of game developers—a figure that has shown little progress in recent years (Game Developers Conference, 2024). The gender disparity is even more pronounced in leadership roles, where 87% of developers with over 20 years of experience are men. This highlights a significant structural imbalance within the industry. This lack of diversity in top-tier roles not only hinders inclusion but also limits innovation and perspectives within the gaming sector (Women in Games, 2024). This is not a new issue; in the past, critical voices have often accused the game production field of fostering toxic work environments for women, highlighting the urgent need for significant improvements (Harvey and Fisher, 2015; Hepler, 2019).

## Conclusion

Like the first version of Poder Violeta, the second iteration of this game demonstrated that video games with a critical focus serve as instruments for social change, fostering empathy and critical reflection on intricate social issues while challenging media representations of certain topics. In this sense, critical video games can be seen as a form of art aimed at promoting a more just and equitable culture. Additionally, the interactive nature of video games makes them a powerful tool for engaging players in the exploration of social and political issues, fostering awareness and encouraging civic action.

In the case of Poder Violeta 2, this experiment underscored the value of iteration in the design process, particularly when the active participation of individuals directly influenced the development of key game elements. Participants not only contributed to the creation of characters and their attributes, but also shaped other fundamental aspects, such as the integration and design of power-ups, the fine-tuning of gameplay dynamics, and the adjustment of game mechanics to better align with the overarching narrative goals. These collaborative interventions enriched the game’s storytelling and enhanced its immersive qualities, making the narrative more engaging and impactful for players.

Furthermore, this iterative process also revealed the importance of co-design in critical video game development, as it allows for a diverse range of perspectives to be incorporated into the creative process. By involving participants in meaningful decision-making, the game achieved a deeper connection with its intended audience, as the collaborative design choices were more reflective of the social issues the game sought to address.

This experience is a valuable reference to feminism media studies, and participatory game design by integrating marginalized voices throughout all design stages and employing procedural rhetoric to embed feminist critiques directly into gameplay mechanics. However, it is necessary to critically acknowledge the limitations of video games as tools for social change. While Poder Violeta 2 fosters critical reflection and dialogue, its impact is mediated by broader cultural and structural factors beyond the game itself. Furthermore, although the game specifically addresses sexual harassment in Bogotá’s public transport, its adaptable framework offers potential for expansion to other forms of harassment and diverse urban contexts, thereby broadening its relevance and applicability.

Finally, as can be inferred, increasing the presence of women and sexually dissident individuals in development teams is not just a desirable goal but an essential challenge for the video game industry. Diverse teams bring a broader range of perspectives, lived experiences, and creative approaches, which are critical for designing games that resonate with varied audiences and challenge existing social norms. By fostering inclusion, the industry can break away from homogenized narratives and stereotypical representations that often dominate mainstream games, creating instead richer, more nuanced storytelling that reflects the complexity of human experiences.

This transformation within the industry is also a critical step toward fostering the development of socially transformative video games that can act as tools for cultural and systemic change. Games designed by diverse teams are better equipped to address intersectional issues and encourage empathy, dialogue, and action. Ultimately, this shift can redefine the role of video games in society—not just as a form of entertainment but as a medium with the potential to contribute to meaningful social progress.

## Data Availability

The raw data supporting the conclusions of this article will be made available by the authors, without undue reservation.
